# Supplementary figures and images for: Simultaneous coherent structure coloring facilitates interpretable clustering of scientific data by amplifying dissimilarity
Source: PLoS One. 2019 Mar 13;14(3):e0212442. doi: 10.1371/journal.pone.0212442 (PMC6415781; doi:10.1371/journal.pone.0212442)

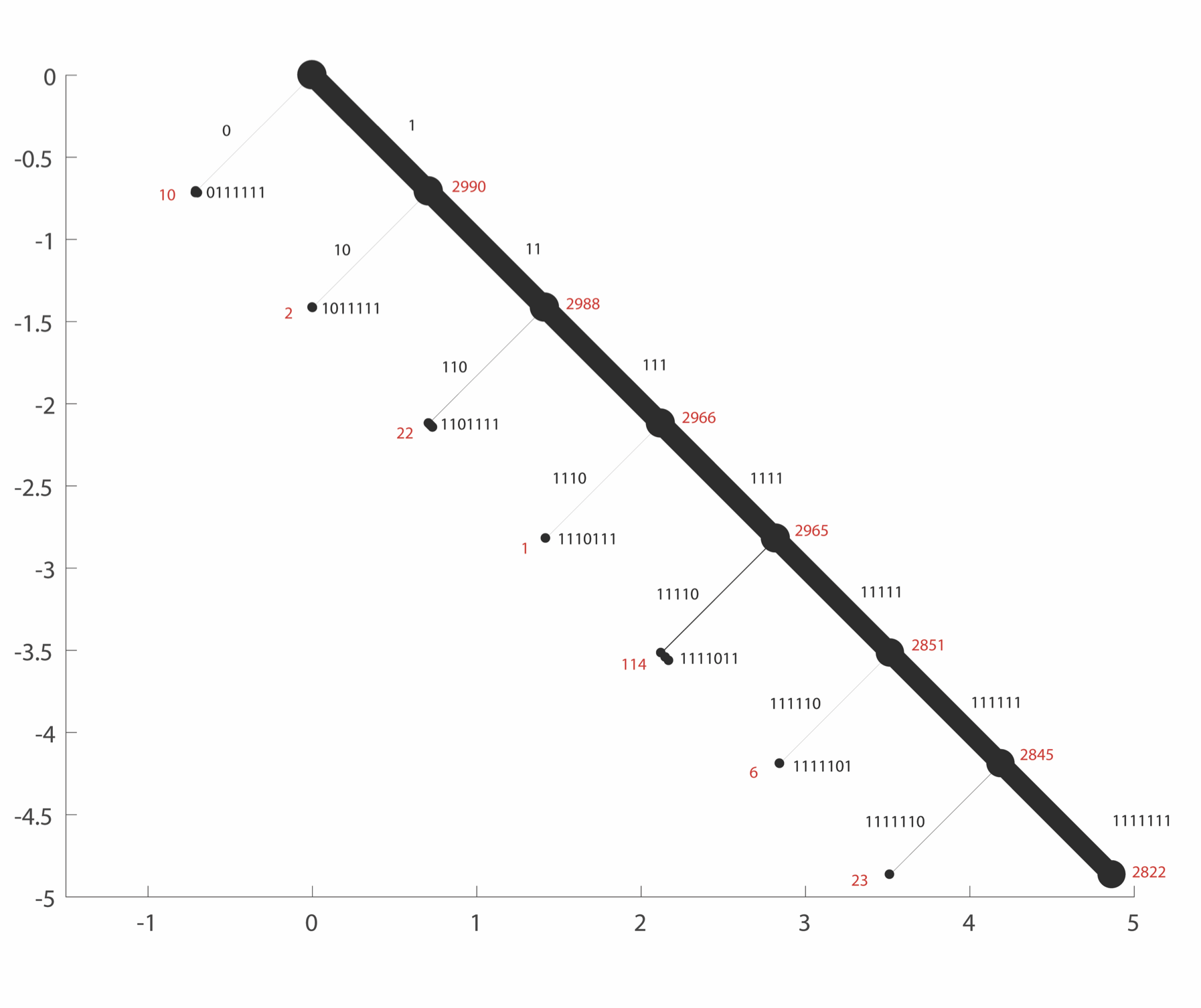

Supplement: S1 Fig — To evaluate the sCSC dendrogram structure resulting from random noise, an adjacency matrix was constructed based on 3000 two-dimensional trajectories whose instantaneous positions over 2000 time steps were selected randomly from uniform distributions over the spatial coordinate intervals x = (0, 1), y = (0, 1). These states were analyzed using pairwise dissimilarity based on the normalized standard deviation. The result is a single main branch with a small splintering of trajectories at each eigenvector level. The splintering at each level converges throughout the seven eigenvectors included in the analysis. The width of each branch is proportional to the fraction of the states that it contains. The corresponding binary code of each branch is labeled in black text, and the number of trajectories associated with each node is labeled in red text. (TIFF) [file pone.0212442.s001.tiff]
