# Supplementary material for: Simultaneous coherent structure coloring facilitates interpretable clustering of scientific data by amplifying dissimilarity
Source: PLoS One. 2019 Mar 13;14(3):e0212442. doi: 10.1371/journal.pone.0212442 (PMC6415781; doi:10.1371/journal.pone.0212442)
Supplement: S1 Table — Summary statistics for the nine macrostates identified from the sCSC model of Protein G. The first column identifies the branch label in Fig 6. The second column shows the number of the original 175 MSM states contained in the macrostate. For the next three columns, statistics are gathered using the one sampled state from each macrostate that is used for visualization in Fig 6. The third column reports the average ± one standard deviation pairwise RMSD over all atoms for all pairs of original MSM states within the same macrostate (e.g. the average RMSD for Branch 0, which contains 29 microstates, each of which is represented by one sampled structure from each original MSM state, is the average of the pairwise RMSD for (292)=406 possible pairs). The fourth and fifth columns report the average percentage ± one standard deviation of the α-helical and β-sheet secondary structure for each original MSM structure sample in the macrostate according to the simplified dictionary of protein secondary structure (DSSP) protocol [61] implemented in the MDTraj [62] software package. The last column indicates which Protein G residues were aligned to create the superpositions illustrated in Fig 6 for each branch. (PDF) [file pone.0212442.s002.pdf]

Branch	Population	Pairwise RMSD (Å)	% α-helical	% β-sheet	Aligned residues
0	29	2.35 ± 0.34	25.0 ± 3.2	43.7 ± 2.7	1–56
1010	11	7.78 ± 2.04	20.3 ± 6.1	34.1 ± 4.0	2–41
1011	22	9.63 ± 2.27	17.5 ± 7.7	31.1 ± 5.6	10–40
110	20	11.95 ± 1.96	4.0 ± 4.1	36.1 ± 6.9	8–24
1110	13	12.01 ± 2.14	5.2 ± 8.4	32.1 ± 5.9	1–39
11110	11	12.06 ± 1.93	24.0 ± 10.4	5.7 ± 8.9	29–45
111110	10	11.29 ± 1.85	8.7 ± 7.0	32.0 ± 10.8	11–37
1111110	12	10.59 ± 2.11	12.9 ± 6.3	33.0 ± 10.8	1–35
1111111	47	11.28 ± 2.40	14.1 ± 8.6	28.7 ± 10.2	1–37
Total	175	10.76 ± 2.85	15.1 ± 9.9	31.9 ± 11.3	-
